# Adult-Onset Deletion of CDKL5 in Forebrain Glutamatergic Neurons Impairs Synaptic Integrity and Behavior in Mice

**DOI:** 10.3390/ijms26146626

**Published:** 2025-07-10

**Authors:** Nicola Mottolese, Feliciana Iannibelli, Giulia Candini, Federica Trebbi, Manuela Loi, Angelica Marina Bove, Giorgio Medici, Zhi-Qi Xiong, Elisabetta Ciani, Stefania Trazzi

**Affiliations:** 1 Department of Biomedical and Neuromotor Sciences, University of Bologna, 40126 Bologna, Italy; nicola.mottolese2@unibo.it (N.M.); feliciana.iannibell2@unibo.it (F.I.); giulia.candini4@unibo.it (G.C.); federica.trebbi3@unibo.it (F.T.); manuela.loi3@unibo.it (M.L.); angelicamarina.bove2@unibo.it (A.M.B.); giorgio.medici2@unibo.it (G.M.); 2 State Key Laboratory of Neuroscience, Institute of Neuroscience, CAS Center for Excellence in Brain Science and Intelligence Technology, Shanghai 200031, China; xiongzhiqi@ion.ac.cn

**Keywords:** CDKL5 deficiency disorder, postdevelopmental stage, conditional knockout mouse models, glutamatergic neurons, behavioral phenotyping

## Abstract

Cyclin-dependent kinase-like 5 (CDKL5) deficiency disorder (CDD) is a severe X-linked neurodevelopmental condition characterized by early-onset, intractable epilepsy, motor and cognitive impairment, and autistic-like features. Although constitutive *Cdkl5* knockout (KO) models have established the importance of CDKL5 during early brain development, CDKL5’s role in the mature brain remains poorly defined. Here, we employed an inducible, conditional KO model in which *Cdkl5* is selectively deleted from forebrain glutamatergic neurons in adult mice to investigate the postdevelopmental functions of CDKL5. Using a total of 48 adult male mice, including *Cdkl5^flox/Y^*(Cre^+^) (*n* = 30) and *Cdkl5^flox/Y^*(Cre^−^) littermate controls (*n* = 18), we found that tamoxifen-induced *Cdkl5* deletion led to prominent behavioral impairments, including deficits in motor coordination, reduced sociability, and impaired hippocampus-dependent spatial memory, while behavioral features such as hyperactivity and stereotypic jumping, typically present in germline KOs, were absent. Sensory functions, including olfaction and pain perception, were also preserved. At the cellular level, the loss of Cdkl5 resulted in a marked reduction in excitatory synapse density in the cortex and hippocampus, accompanied by increased numbers of immature dendritic spines and decreased mature spines. Neuronal loss in the hippocampal CA1 region and selective microglial activation in the cortex were also observed. These alterations closely resemble those seen in constitutive KO models, underscoring the ongoing requirement for CDKL5 expression in excitatory neurons for maintaining synaptic integrity and neuronal homeostasis in the adult brain. This study underscores the importance of temporally controlled models for investigating the mechanisms underlying CDD pathophysiology in the adult brain.

## 1. Introduction

Variants in the X-linked cyclin-dependent kinase-like 5 (*CDKL5*) gene are associated with a rare neurodevelopmental disorder known as CDKL5 deficiency disorder (CDD) [1,2,3,4,5,6,7,8,9]. The *CDKL5* gene encodes a serine/threonine kinase [10,11,12,13] that is abundantly expressed in both the developing and adult brain [14,15], and plays a crucial role in regulating key processes essential for proper neural network formation [16,17,18,19,20,21,22]. The loss of CDKL5 function leads to a complex clinical phenotype, characterized by early infantile-onset and drug-resistant epilepsy, followed by severe neurodevelopmental impairment [23,24,25,26,27,28,29,30,31]. Associated neurological features include hypotonia, intellectual and motor disabilities, autistic-like traits, and cortical visual impairment, as well as other comorbidities such as gastrointestinal issues and sleep disturbances [32,33,34,35,36,37,38,39,40,41].

Over the past several years, multiple constitutive *Cdkl5* knockout (KO) and knockin (KI) mouse models have been generated by permanently inactivating *Cdkl5* in all cells and tissues throughout development [42,43,44,45,46,47]. These animal models, which exhibit constitutive Cdkl5 deficiency, recapitulate most of the phenotypic features of the disorder, including severe impairments in hippocampal-dependent learning and memory, autistic-like behaviors, altered social interactions, visual and respiratory deficits, and motor stereotypies [42,43,45,48,49,50,51,52,53]. Neuroanatomical analyses have shown that these defects are associated with altered neuronal architecture, characterized by impairments in dendritic arborization, synaptogenesis, and the connectivity of pyramidal neurons in both the cortical [54,55,56,57] and hippocampal regions [43,48,50] of *Cdkl5* KO mice. Additionally, the absence of Cdkl5 leads to decreased survival of CA1 pyramidal neurons in the hippocampus of these animal models [48,58], as well as the presence of a generalized neuroinflammatory process [59].

Although most studies on *Cdkl5* KO mice have primarily focused on the adult brain phenotype that results from constitutive inactivation, a recent comparative analysis of brain defects across developmental stages in a *Cdkl5* KO mouse model has shown that many structural alterations emerge early in postnatal development and are largely maintained throughout later stages of brain maturation [57]. These findings suggest that CDKL5 function is crucial for early postnatal developmental processes that underlie brain maturation. However, using a genetic approach to selectively ablate *Cdkl5* expression in adult mice, Terzic and colleagues demonstrated that the postdevelopmental deletion of *Cdkl5* leads to behavioral and neuroanatomical defects similar to those observed in germline knockout mice [60], suggesting that CDKL5 also plays a crucial functional role in the mature brain.

Since Cdkl5 is expressed in both glutamatergic and GABAergic neurons [14,61,62], conditional knockout (cKO) models have been developed to selectively delete *Cdkl5* in forebrain excitatory [43,44,62,63] or inhibitory neurons [43,62], in order to identify the specific cell type in which Cdkl5 deficiency drives pathological phenotypes. These studies highlight the critical role of CDKL5 in glutamatergic neurons, where it serves as a key regulator of circuit development and synaptic activity. Notably, the prenatal deletion of *Cdkl5* in forebrain excitatory neurons leads to both morphological and functional changes in hippocampal and cortical neurons, impairing synaptic activity, hippocampal-dependent memory, and cortical visual responses [44,63].

Although conditional knockout (cKO) mouse models have been pivotal in advancing our understanding of the primary role of Cdkl5 in glutamatergic neurons during brain development, the selective deletion of *Cdkl5* in these neurons within the mature brain has yet to be investigated. Understanding how the absence of CDKL5 in the adult brain impacts the brain phenotypes is crucial in order to predict the effectiveness of future therapies aimed at restoring CDKL5 expression in CDD patients. To investigate the role of CDKL5 in the adult brain, we used a recently developed cKO mouse model that allows for the inducible deletion of *Cdkl5* expression in forebrain glutamatergic neurons [61,64]. Our findings demonstrate that the loss of *Cdkl5* in excitatory neurons of the adult brain leads to neuroanatomical alterations, including a reduction in excitatory synapse density, impaired dendritic spine maturation, and decreased neuronal survival. These structural changes were accompanied by increased microglial activation, indicative of localized neuroinflammation. Behaviorally, *Cdkl5* cKO mice exhibited impairments in hippocampus-dependent learning and memory, reduced sociability, motor coordination deficits, and autistic-like features. Collectively, these results highlight that CDKL5 plays a critical postdevelopmental role in glutamatergic neurons, maintaining the synaptic architecture, neuronal viability, and functional integrity of forebrain circuits essential for normal cognitive and social behaviors.

## 2. Results

### 2.1. Postnatal Cdkl5 Deletion in Forebrain Glutamatergic Neurons, and Body Weight and General Health in Cdkl5^flox/Y^(Cre^+^) Mice

**Figure 1:**
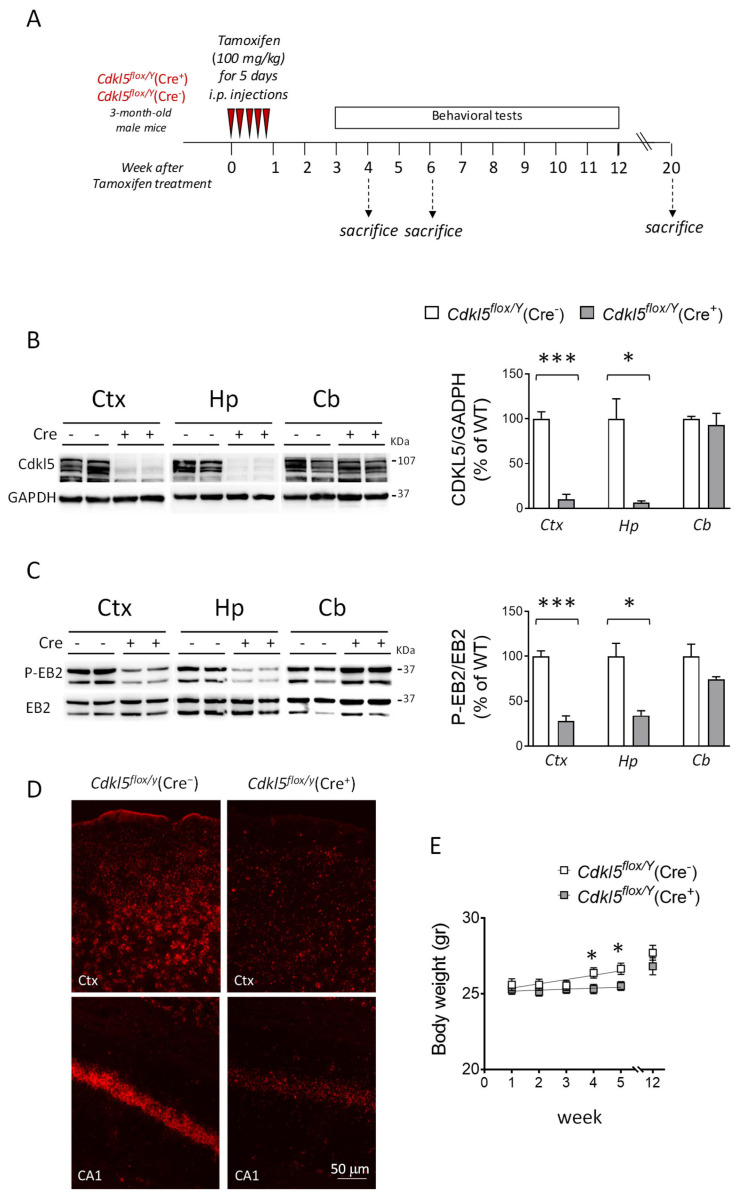
Postnatal *Cdkl5* deletion in forebrain glutamatergic neurons of *Cdkl5^flox/Y^*(Cre^+^) mice. (**A**) A schematic representation of the experimental design. Three-month-old conditional knockout (cKO) hemizygous male *Cdkl5^flox/Y^*(Cre^+^) mice received intraperitoneal (i.p.) injections of tamoxifen (100 mg/kg) for five consecutive days to activate Cre recombinase and induce *Cdkl5* gene deletion. Their *Cdkl5^flox/Y^*(Cre^−^) littermates were used as controls. The animals were divided into three groups and sacrificed at different time points (4, 6, and 20 weeks after the start of tamoxifen treatment). (**B**) Western blot analysis of Cdkl5 protein expression in tamoxifen-treated mice. Protein extracts from the somatosensory cortex (Ctx), hippocampus (Hp), and cerebellum (Cb) were analyzed in *Cdkl5^flox/Y^*(Cre^−^) (*n* = 3) and *Cdkl5^flox/Y^*(Cre^+^) (*n* = 3) mice. The histogram on the right shows Cdkl5 levels normalized to GAPDH and expressed as a percentage relative to the controls. Representative immunoblots for Cdkl5 and GAPDH from two animals per group are shown on the left. (**C**) Western blot analysis of phosphorylated EB2 (phospho-EB2) and total EB2. Protein extracts from the cortex (Ctx), hippocampus (Hp), and cerebellum (Cb) were analyzed as in panel (**B**). The histogram on the right shows phospho-EB2 levels normalized to total EB2 and expressed relative to the controls. Representative immunoblots from two animals per group are shown on the left. The values in panels (**B**,**C**) are presented as means ± SEM. * *p* < 0.05, *** *p* < 0.001 (two-tailed Student’s *t*-test). (**D**) Representative fluorescence images of cortical and hippocampal sections from one animal per group processed by in situ hybridization (ISH) for *Cdkl5* mRNA (red). Scale bar = 50 µm. (**E**) Body weight (in grams) of *Cdkl5^flox/Y^*(Cre^−^) (*n* = 15–18) and *Cdkl5^flox/Y^*(Cre^+^) (*n* = 23–26) mice measured weekly over a five-week period following tamoxifen treatment, and of an additional cohort of *Cdkl5^flox/Y^*(Cre^−^) (*n* = 15) and *Cdkl5^flox/Y^*(Cre^+^) (*n* = 10) mice assessed 12 weeks after tamoxifen administration. The data are shown as the mean ± SEM. * *p* < 0.05, Fisher’s LSD post hoc test following two-way ANOVA. Abbreviations: Ctx = somatosensory cortex; Hp = hippocampus; Cb = cerebellum; CA1 = hippocampal CA1 region.

To investigate whether *Cdkl5* deletion in excitatory neurons of the adult brain leads to neurobehavioral alterations, we used a previously established tamoxifen-inducible conditional knockout (cKO) *Cdkl5* mouse model [61,64]. Three-month-old cKO male mice carrying an inducible Cre recombinase gene (CamK2α-iCre) under the control of the mouse *CamK2α* (calcium/calmodulin-dependent protein kinase 2 alpha) promoter, hereafter referred to as *Cdkl5^flox/Y^*(Cre^+^) mice, and controls comprising their Cre-negative male littermates (*Cdkl5^flox/Y^*(Cre^−^)), were administered tamoxifen via intraperitoneal injection for five consecutive days to induce the postnatal, forebrain-specific deletion of *Cdkl5* in glutamatergic neurons (Figure 1A). Mice were subjected to a series of behavioral tests between the third and twelfth weeks following tamoxifen treatment. These tests evaluated locomotor activity and coordination, spontaneous home-cage behaviors, sociability, hippocampal-dependent learning and memory, and sensory abilities (Figure 1A). Mice were sacrificed four weeks after the initiation of treatment to confirm the inactivation of the *Cdkl5* gene, or after six or twenty weeks to evaluate the presence of neuroanatomical changes and their temporal progression (Figure 1A).

To assess the effective inactivation of the *Cdkl5* gene, we analyzed the expression levels of Cdkl5 and the phosphorylation status of its well-known target, EB2 [65], in brain extracts from *Cdkl5^flox/Y^*(Cre^+^) and *Cdkl5^flox/Y^*(Cre^−^) mice through Western blot. A significant decrease in Cdkl5 expression was observed in homogenates from the cortical and hippocampal tissue of *Cdkl5^flox/Y^*(Cre^+^) mice compared to their littermate *Cdkl5^flox/Y^*(Cre^−^) controls. In contrast, Cdkl5 levels remained unchanged in cerebellar tissue, confirming the specific knockout of *Cdkl5* in forebrain excitatory neurons (Figure 1B). Accordingly, a decrease in Cdkl5 kinase activity was observed in the cortex and hippocampus of *Cdkl5^flox/Y^*(Cre^+^) mice, as evidenced by the reduced phosphorylation levels of EB2 (Figure 1C).

To further validate the forebrain-specific deletion of *Cdkl5*, in situ hybridization for *Cdkl5* mRNA was performed on brain sections from *Cdkl5^flox/Y^*(Cre^+^) and Cre^−^ mice (Figure 1D and Figure S1). Although fluorescence intensity was not quantitatively measured, a marked reduction in the *Cdkl5* mRNA signal was clearly observed in the cortex and hippocampus of Cre^+^ mice compared to the Cre^−^ controls (Figure 1D and Figure S1). In contrast, the *Cdkl5* mRNA levels remained unchanged in other brain regions, including the striatum, thalamus, and cerebellum (Figure S1). This regional expression pattern confirms the selective deletion of *Cdkl5* in forebrain glutamatergic neurons and aligns with the known higher density of excitatory neurons in the cortex and hippocampus [66,67].

Regarding animal welfare, *Cdkl5^flox/Y^*(Cre^+^) mice exhibited a slight reduction in body weight compared to the Cre^−^ controls during the fourth and fifth weeks following tamoxifen administration. Although this reduction persisted up to 12 weeks, it did not reach statistical significance (Figure 1E), suggesting that Cdkl5 deficiency may modestly impair somatic growth or influence food intake. Nonetheless, no signs of compromised welfare were detected in *Cdkl5^flox/Y^*(Cre^+^) mice, as indicated by their normal fur condition, unimpaired mobility within their home cage, and absence of spontaneous spasms.

The absence of spontaneous epileptic events, although differing from previous observations in this model [61,64], allowed us to perform a comprehensive series of behavioral analyses. Since the constitutive loss of *Cdkl5* in *Cdkl5* KO mice has been associated with a broad range of behavioral impairments, including deficits in motor coordination, autistic-like traits, reduced social interactions, and memory impairments [42,43,68,69,70], we assessed these same behavioral domains in the *Cdkl5^flox/Y^*(Cre^+^) cKO mouse model.

### 2.2. Motor Coordination, Repetitive and Autistic-like Behaviors in Cdkl5^flox/Y^(Cre^+^) Mice

**Figure 2:**
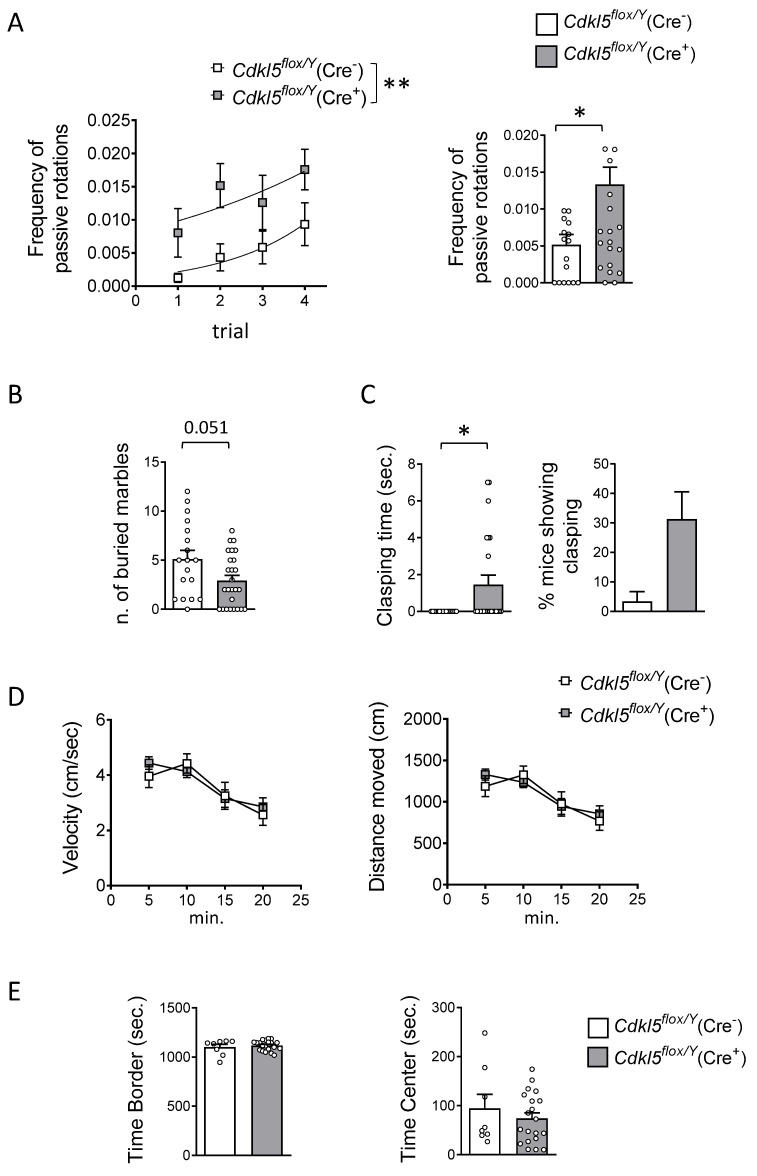
The effect of postnatal *Cdkl5* deletion in forebrain glutamatergic neurons on motor control and autistic-like behaviors in *Cdkl5^flox/Y^*(Cre^+^) mice. (**A**) The accelerating rotarod test performed over four consecutive trials with 1 h inter-trial intervals. The graph on the left shows the number of passive rotations per trial (i.e., instances in which the mouse remains immobile and is passively carried by the rotating rod). The graph on the right reports the average number of passive rotations across trials in tamoxifen-treated *Cdkl5^flox/Y^*(Cre^−^) (*n* = 17) and *Cdkl5^flox/Y^*(Cre^+^) (*n* = 26) mice. (**B**) The assessment of repetitive and stereotyped behaviors using the marble-burying test. The number of marbles buried by *Cdkl5^flox/Y^*(Cre^−^) (*n* = 18) and *Cdkl5^flox/Y^* (Cre^+^) (*n* = 26) mice is shown. (**C**) Hind-limb clasping behavior: the total duration of clasping behavior over a 2 min observation period (left) and the percentage of mice displaying clasping (right) in tamoxifen-treated *Cdkl5^flox/Y^*(Cre^−^) (*n* = 16) and *Cdkl5^flox/Y^*(Cre^+^) (*n* = 24) mice. (**D**) Locomotor activity assessed in a 20 min open field test. The graphs show the average locomotion velocity (left) and total distance traveled (right) in tamoxifen-treated *Cdkl5^flox/Y^*(Cre^−^) (*n* = 8) and *Cdkl5^flox/Y^*(Cre^+^) (*n* = 20) mice. (**E**) Anxiety-like behavior in the open field test: time spent in the border (left) and center (right) zones of the arena during the same 20 min session as in panel (**D**). The data are presented as the mean ± SEM. * *p* < 0.05, ** *p* < 0.01. Unpaired two-tailed Student’s *t*-test for data in (**E**); Mann–Whitney test for data in (**A**) right graph, (**B**), and (**C**); Fisher’s LSD post hoc test following two-way ANOVA for (**A**) left graph and (**D**).

We evaluated motor coordination using an accelerating rotarod assay by analyzing the frequency of passive rotations (number of passive rotations per second). Locomotor performance was significantly impaired in *Cdkl5^flox/Y^*(Cre^+^) mice compared to *Cdkl5^flox/Y^*(Cre^−^) control mice (genotype: F(1, 160) = 11.11, *p* = 0.0011; two-way ANOVA), as evidenced by the higher number of passive rotations observed in all subsequent testing trials (Figure 2A).

In the marble burying assay, used to assess autistic-like behavior, *Cdkl5^flox/Y^*(Cre^+^) mice exhibited significantly impaired behavior, burying fewer marbles compared to their (Cre^−^) counterparts (Figure 2B).

As a sign of stereotypic movements, we assessed the clasping time in *Cdkl5^flox/Y^*(Cre^+^) mice. We found that *Cdkl5^flox/Y^*(Cre^+^) mice exhibited increased clasping of the hind-limbs, as evidenced by a longer duration of sustained clasping and a higher percentage of mice displaying this phenotype compared to *Cdkl5^flox/Y^*(Cre^−^) control mice (Figure 2C).

The open field test was employed to evaluate both locomotor activity and anxiety-like behavior. In contrast to constitutive *Cdkl5* KO mice, which display hyperactivity in this assay [42,43,69], *Cdkl5^flox/Y^*(Cre^+^) mice exhibited normal activity levels compared to the *Cdkl5^flox/Y^*(Cre^−^) controls, with no significant differences in either velocity or total distance traveled (Figure 2D). Both groups showed a progressive decrease in locomotor activity over repeated trials, suggesting habituation to the environment (time: F(3, 104) = 9.073, *p* < 0.0001; two-way ANOVA). With regard to the stereotypic jumping behavior that characterizes constitutive *Cdkl5* KO mice, we did not observe this phenotype in *Cdkl5^flox/Y^*(Cre^+^) animals under our testing conditions, suggesting that this specific behavioral trait may depend on the early developmental loss of Cdkl5 rather than from its postnatal ablation in forebrain excitatory neurons. To assess anxiety-like behavior, we compared the time spent in the peripheral versus central zones of the arena and found no significant differences between groups (Figure 2E).

### 2.3. Social Interaction and Memory Impairments in Cdkl5^flox/Y^(Cre^+^) Mice

**Figure 3:**
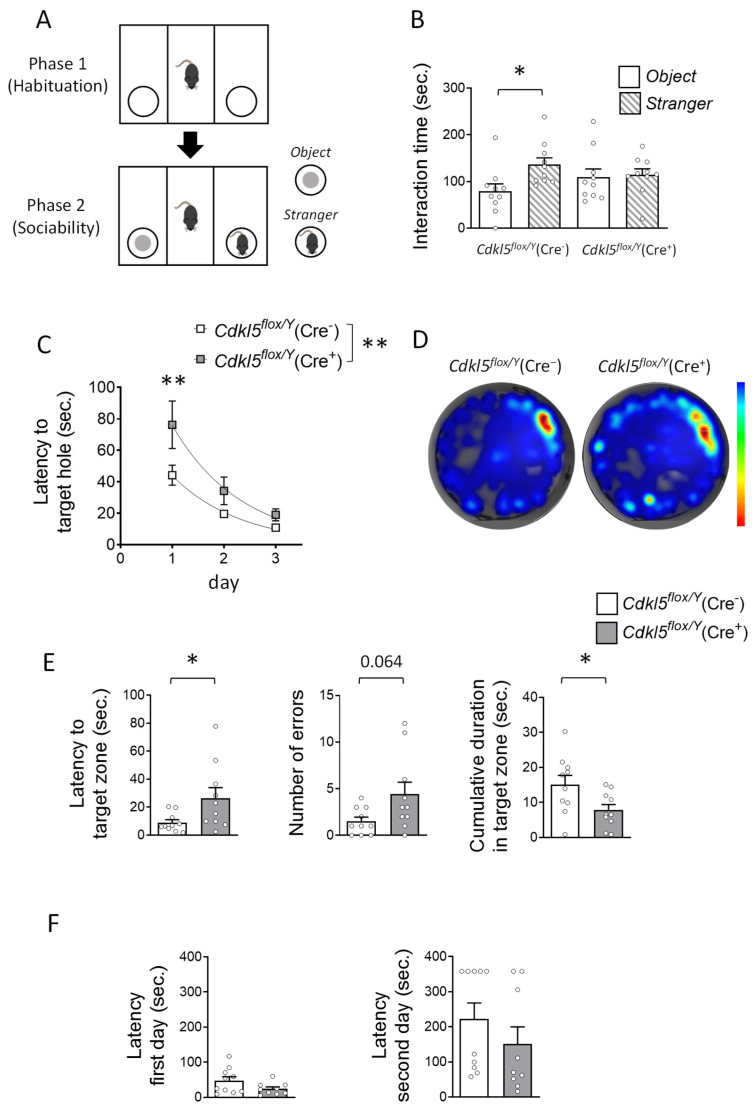
The effects of postnatal *Cdkl5* deletion in forebrain glutamatergic neurons on sociability and hippocampal-dependent memory in *Cdkl5^flox/Y^*(Cre^+^) mice. (**A**,**B**) The Three-Chamber Social Interaction Test. Panel (**A**) illustrates a schematic of the experimental paradigm. Panel (**B**) quantifies the cumulative time spent interacting with either an inanimate object or a social partner during the second trial (sociability phase). The test was conducted on the same cohort of tamoxifen-treated *Cdkl5^flox/Y^*(Cre^−^) (*n* = 10) and *Cdkl5^flox/Y^*(Cre^+^) (*n* = 10) mice. The asterisk denotes within-subject comparison. The data are presented as the mean ± SEM. * *p* < 0.05, Fisher’s LSD post hoc test following two-way ANOVA. (**C**–**E**) Barnes maze—spatial learning and memory. Spatial learning performance during the 3-day acquisition phase is shown in Panel (**C**) as the mean latency to find the target hole. Panel (**D**) presents representative heat maps from the probe trial (day 4), with warmer colors indicating longer dwell times. Panel (**E**) displays the quantitative results from the probe trial, including (left) the latency to enter the target zone, (middle) the number of errors (incorrect hole entries), and (right) the cumulative time spent in the target zone. (**F**) Passive avoidance test. The graph depicts the latency to enter the dark compartment on day 1 (training) and day 2 (retention) for tamoxifen-treated *Cdkl5^flox/Y^*(Cre^−^) and *Cdkl5^flox/Y^*(Cre^+^) mice (*n* = 10 and 9 per group, respectively). The data are presented as the mean ± SEM. * *p* < 0.05, ** *p* < 0.01. Fisher’s LSD post hoc test following two-way ANOVA (**C**); unpaired two-tailed Student’s *t*-test ((**B**) within groups, (**E**) right graph, and **F**); Welch’s *t*-test ((**E**) left graph); Mann–Whitney test ((**E**) middle graph).

To assess social interaction in *Cdkl5^flox/Y^*(Cre^+^) mice, we performed the three-chamber test (Figure 3A), which quantifies sociability by measuring the time spent interacting with a novel mouse versus a non-social object. While *Cdkl5^flox/Y^*(Cre^−^) control mice showed a clear preference for the stranger mouse over the object, *Cdkl5^flox/Y^*(Cre^+^) mice failed to display such a preference, spending comparable amounts of time with both the mouse and the object (Figure 3B). These results indicate impaired social interaction in the *Cdkl5^flox/Y^*(Cre^+^) group.

To evaluate hippocampus-dependent learning and memory, we employed the Barnes maze and passive avoidance tests. During the learning phase of the Barnes maze, mice were trained to locate a hidden escape box using spatial cues positioned around the arena. *Cdkl5^flox/Y^*(Cre^+^) mice demonstrated the ability to learn the task over time; however, their learning curve was consistently higher compared to that of *Cdkl5^flox/Y^*(Cre^−^) control mice (genotype: F(1, 54) = 8.201, *p* = 0.0059; two-way ANOVA), indicating slower acquisition of the task (Figure 3C). When daily performance was compared between groups using Fisher’s LSD test, a significant difference emerged on the first day of training (*p* = 0.0053), while differences on subsequent days did not reach statistical significance (Figure 3C). In the day 4 probe test, *Cdkl5^flox/Y^*(Cre^+^) mice exhibited significantly impaired memory performance compared to the *Cdkl5^flox/Y^*(Cre^−^) controls. Specifically, they showed a longer latency to locate the former position of the escape box, made a higher number of incorrect hole entries, and spent less time in the target zone overall (Figure 3D,E).

In the passive avoidance test, mice were conditioned with a single aversive stimulus, and their ability to avoid the associated environment was subsequently assessed. No significant differences were observed between *Cdkl5^flox/Y^*(Cre^+^) and *Cdkl5^flox/Y^*(Cre^−^) mice in latency to enter the dark compartment during the training session (Day 1; Figure 3F). In the retention test (Day 2), a trend toward reduced latency was observed in the Cre^+^ group, although this difference did not reach statistical significance, probably due to high inter-individual variability in this test (Figure 3F).

### 2.4. Olfaction and Pain Perception in Cdkl5^flox/Y^(Cre^+^) Mice

**Figure 4:**
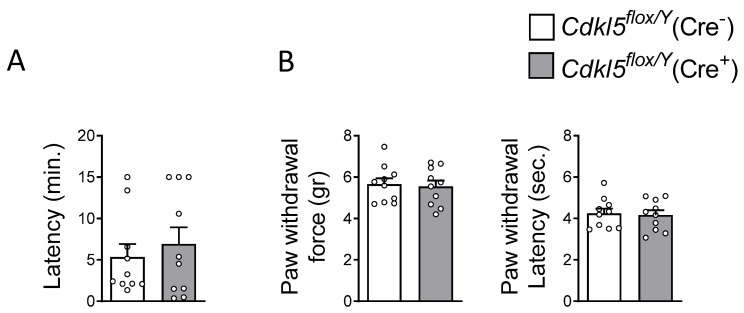
The effects of postnatal *Cdkl*5 deletion in forebrain glutamatergic neurons on olfactory and pain perception in *Cdkl5^flox/Y^*(Cre^+^) mice. (**A**) Olfactory function was assessed using the buried-food test. The histogram shows the latency to locate a buried raisin in tamoxifen-treated *Cdkl5^flox/Y^*(Cre^−^) (*n* = 10) and *Cdkl5^flox/Y^*(Cre^+^) (*n* = 10) mice. (**B**) Pain sensitivity was evaluated using the Von Frey filament test in the same animals. Mechanical allodynia was quantified by measuring the force (in grams) required to elicit paw withdrawal (left) and the latency of hind-paw withdrawal following stimulation (right). The data are presented as the mean ± SEM. Unpaired two-tailed Student’s *t*-test.

To determine whether the loss of *Cdkl5* from excitatory forebrain neurons in adulthood affects sensory abilities due to impaired central sensory signal integration, we assessed olfactory function and pain perception in *Cdkl5^flox/Y^*(Cre^+^) mice. Olfactory performance was tested using the buried-food test, which evaluates the animal’s ability to locate hidden food using olfactory cues. No significant difference was observed between *Cdkl5^flox/Y^*(Cre^+^) and *Cdkl5^flox/Y^*(Cre^−^) mice (Figure 4A), indicating intact olfactory function in the Cre^+^ group.

Given that altered pain perception has been reported in CDD patients and in constitutive *Cdkl5* KO mice [71], we next evaluated mechanical nociception using the Von Frey filament test. *Cdkl5^flox/Y^*(Cre^+^) and *Cdkl5^flox/Y^*(Cre^−^) mice showed no significant differences in mechanical sensitivity, as reflected by comparable paw withdrawal thresholds and similar latencies of hind-paw withdrawal following stimulation (Figure 4B).

These results suggest that the postnatal deletion of *Cdkl5* in excitatory neurons does not impair basic sensory processing.

### 2.5. Brain Connectivity and Hippocampal Dendritic Spine Maturation in Cdkl5^flox/Y^(Cre^+^) Mice

**Figure 5:**
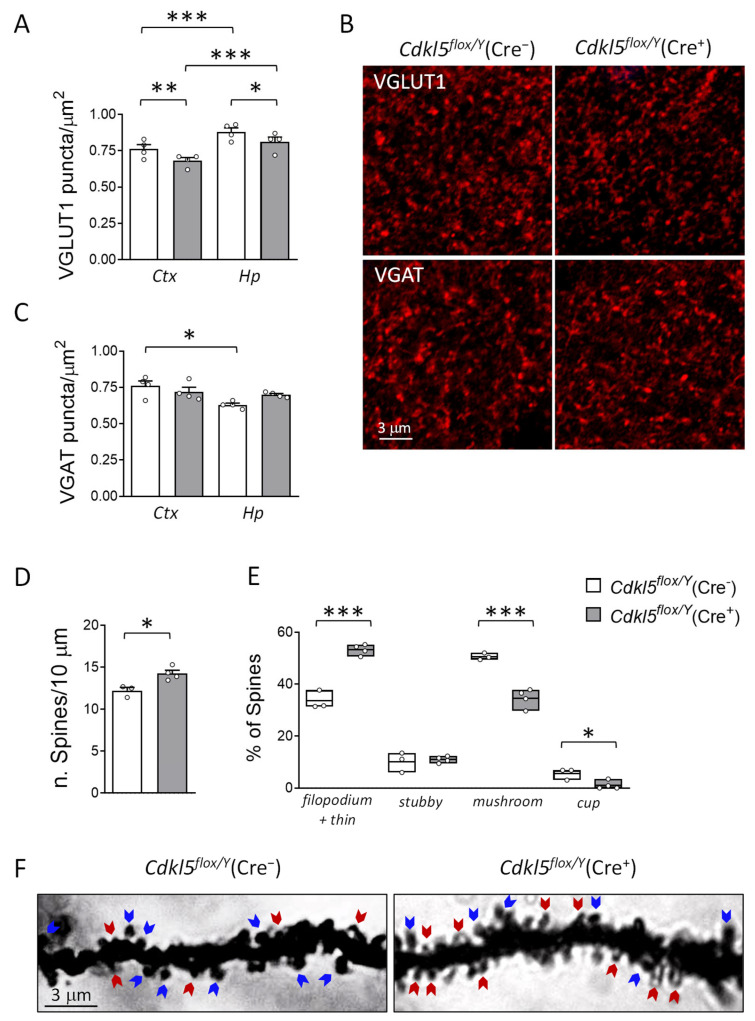
The effects of postnatal *Cdkl5* deletion in forebrain glutamatergic neurons on neuronal connectivity and dendritic spine maturation in *Cdkl5^flox/Y^*(Cre^+^) mice. (**A**,**B**) The quantification of fluorescent puncta per μm^2^ positive for vesicular glutamate transporter 1 (VGLUT1, (**A**)) and vesicular GABA transporter (VGAT, (**B**)) in the somatosensory cortex (Ctx) and hippocampus (Hp) of *Cdkl5^flox/Y^*(Cre^−^) (*n* = 4) and *Cdkl5^flox/Y^*(Cre^+^) (*n* = 4) mice six weeks after tamoxifen treatment. (**C**) Representative confocal images showing VGLUT1 (top) and VGAT (bottom) immunostaining in cortical sections from one mouse per group. Scale bar = 3 μm. (**D**,**E**) Dendritic spine analysis in CA1 pyramidal neurons of the hippocampus of *Cdkl5^flox/Y^*(Cre^−^) (*n* = 3) and *Cdkl5^flox/Y^*(Cre^+^) (*n* = 4) mice twenty weeks after tamoxifen treatment. Panel (**D**) shows the spine density in basal dendrites; panel (**E**) shows the proportion of immature and mature spine types relative to the total number of protrusions. (**F**) Representative Golgi-impregnated dendrites from one mouse per group. Blue arrows indicate mature spines; red arrows indicate immature spines. Scale bar = 3 μm. The data are expressed as the mean ± SEM. * *p* < 0.05, ** *p* < 0.01, *** *p* < 0.001. Fisher’s LSD post hoc test following two-way ANOVA (**A**,**B**,**E**); two-tailed Student’s *t*-test (**D**).

Since behavioral abnormalities in constitutive *Cdkl5* KO mice are associated with neuroanatomical alterations such as impaired connectivity and dendritic spine maturation [55,56,57,70], we investigated synaptic organization in the hippocampus and cortex of *Cdkl5^flox/Y^*(Cre^+^) mice by analyzing the number of excitatory and inhibitory terminals that were immunoreactive for vesicular glutamate transporter-1 (VGLUT1), a marker of glutamatergic axon terminals [72], and vesicular GABA transporter (VGAT), a marker of GABAergic axon terminals [73], respectively. In both the cortex and hippocampus of *Cdkl5^flox/Y^*(Cre^+^) mice, we observed a reduced number of VGLUT1-positive puncta compared to the *Cdkl5^flox/Y^*(Cre^−^) controls (Figure 5A,B; cortex: −10.77 ± 2.81%; hippocampus: −7.71 ± 3.62%), suggesting a decrease in glutamatergic terminal density following *Cdkl5* deletion. In both experimental groups, the density of VGLUT1-positive puncta was significantly higher in the hippocampus compared to the cortex (Figure 5A; Cre^−^: +15.26 ± 3.53%; Cre^+^: +19.21 ± 4.68%), indicating a regional difference in glutamatergic terminal distribution.

Concerning inhibitory terminals, no differences were found in the density of VGAT-positive puncta between *Cdkl5^flox/Y^*(Cre^+^) and *Cdkl5^flox/Y^*(Cre^−^) mice in either the cortex or the hippocampus (Figure 5B,C). However, the comparison of inhibitory terminal density between the cortex and hippocampus in *Cdkl5^flox/Y^*(Cre^−^) mice revealed a significantly lower density in the hippocampus (Figure 5B; −16.81 ± 1.63%), whereas no such difference was observed in *Cdkl5^flox/Y^*(Cre^+^) mice (Figure 5B).

To investigate whether the loss of *Cdkl5* in excitatory neurons of the adult brain affects spine development, we analyzed Golgi-impregnated brain sections and quantified spine density on the dendrites of pyramidal neurons in the hippocampal CA1 region. *Cdkl5^flox/Y^*(Cre^+^) mice exhibited a significantly increased spine density compared to their Cre^−^ counterparts (Figure 5D; +16.81 ± 3.24%). To further investigate potential alterations in dendritic spine development, we quantified dendritic spines based on their morphology and maturation state. As previously described [74], spines were classified as immature (filopodia, thin-shaped, and stubby-shaped) or mature (mushroom- and cup-shaped). Morphological analysis revealed that adult loss of *Cdkl5* in glutamatergic neurons impaired spine maturation in *Cdkl5^flox/Y^*(Cre^+^) mice. Specifically, hippocampal neurons from *Cdkl5^flox/Y^*(Cre^+^) mice displayed a significant reduction in the proportion of mature spines (Figure 5E,F; mushroom: −31.51 ± 3.52%; cup-shaped: −80.45 ± 14.87%) and a corresponding increase in the percentage of immature spines (Figure 5E,F; filopodia plus thin-shaped: +58.41 ± 3.05%) compared to the *Cdkl5^flox/Y^*(Cre^−^) controls.

### 2.6. Microglial Cell Density and Hippocampal Neuronal Survival in the Brains of Cdkl5^flox/Y^(Cre^+^) Mice

Since the loss of *Cdkl5* has recently been shown to promote neuroinflammatory processes in the cortex and hippocampus of constitutive *Cdkl5* KO mice, along with a reduction in neuronal survival [59], we analyzed the number of microglial cells (AIF-1-positive) in the cortical and hippocampal regions of *Cdkl5^flox/Y^(*Cre^+^) mice at two different time points, six and twenty weeks after tamoxifen treatment, to assess whether the persistence of *Cdkl5* deficiency leads to worsening of the phenotype.

**Figure 6:**
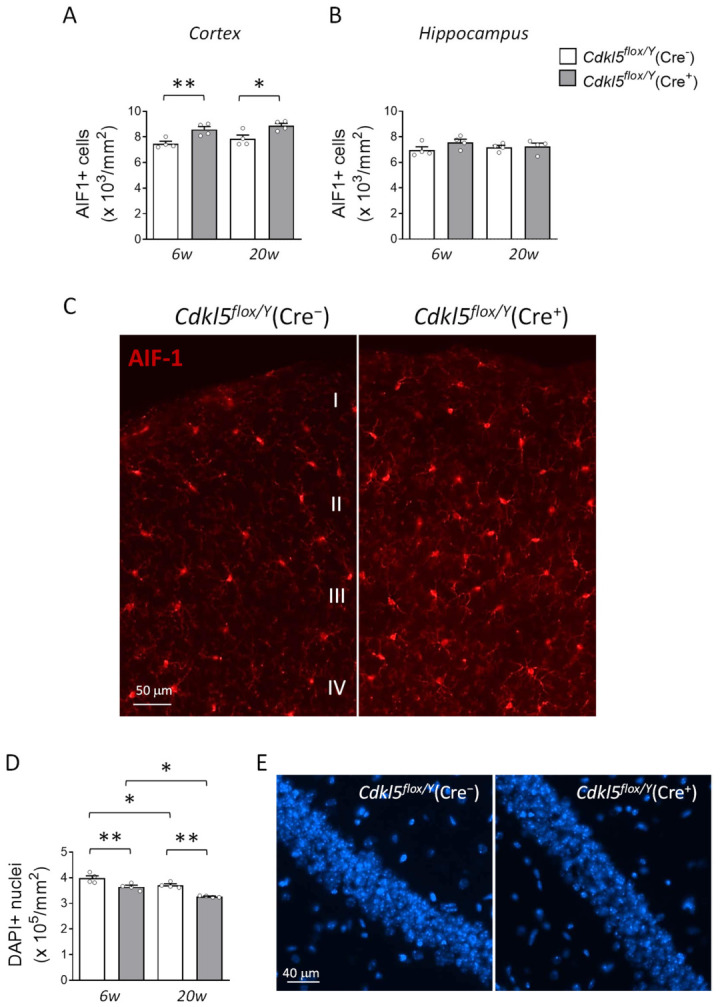
The effects of postnatal *Cdkl5* deletion in forebrain glutamatergic neurons on microglial density and neuronal survival in *Cdkl5^flox/Y^*(Cre^+^) mice. (**A**,**B**) The quantification of AIF-1-positive cells in the somatosensory cortex (**A**) and hippocampus (**B**) of *Cdkl5^flox/Y^*(Cre^−^) and *Cdkl5^flox/Y^*(Cre^+^) mice (*n* = 4 per group) at six (6w) and twenty weeks (20w) after tamoxifen treatment. (**C**) Representative fluorescence images of AIF-1 immunostaining in cortical sections from one mouse per group at six weeks post-treatment. Scale bar = 50 μm. Cortical layers I–IV are indicated. (**D**) The quantification of DAPI-positive cells in the CA1 region of the hippocampus from the same animals analyzed in panel (**B**). (**E**) Representative DAPI-stained hippocampal sections from one mouse per group six weeks post-treatment. Scale bar = 40 μm. The data are shown as the mean ± SEM. * *p* < 0.05, ** *p* < 0.01. Fisher’s LSD post hoc test following two-way ANOVA (**A**,**B**,**D**).

The quantification of AIF-1-positive cells in the somatosensory cortex revealed a higher microglial density in *Cdkl5^flox/Y^*(Cre^+^) mice compared to the Cre^−^ controls as early as six weeks post-treatment (+14.61 ± 3.11%), a difference that persisted at twenty weeks (+12.99 ± 2.37%; Figure 6A,C). In contrast, no significant differences in microglial density were observed between the two groups in the hippocampal region at either time point (Figure 6B).

Regarding hippocampal neuronal survival, *Cdkl5^flox/Y^*(Cre^+^) mice showed a comparable percentage reduction in the number of DAPI-positive nuclei in the CA1 region at both six and twenty weeks following tamoxifen treatment relative to the controls (Figure 6D,E; 6w: −8.38 ± 1.67%, and 20w: −11.90 ± 0.38%). These findings suggest that hippocampal neuron loss occurs early after *Cdkl5* deletion and does not significantly progress over time. In contrast, an age-dependent decline in hippocampal neuron survival was evident in both experimental groups (Figure 6D,E; Cre^−^: −6.87 ± 1.31%, and Cre^+^: −10.45 ± 0.38%).

## 3. Discussion

This study provides compelling evidence for the essential role of CDKL5 in the adult brain, showing that its selective deletion in glutamatergic neurons of the forebrain, even after the completion of brain development, is sufficient to induce neuroanatomical, synaptic, and behavioral alterations that are characteristic of the CDKL5 deficiency disorder (CDD) phenotype. These findings reinforce the hypothesis that CDKL5 is not only critical during early neurodevelopment, but also plays a key role in maintaining neuronal function and the structural integrity of brain circuits throughout adulthood. Previous studies in constitutive *Cdkl5* KO mouse models have highlighted the importance of CDKL5 during early developmental stages, with animals displaying profound synaptic deficits, impaired neuronal maturation, and a wide spectrum of behavioral abnormalities that resemble core features of CDD [42,43,45,48,50,54,55,56,57]. However, it remained unclear whether these phenotypes were exclusively the result of early developmental dysfunction or whether CDKL5 continued to exert essential functions in the mature brain. By employing a conditional and neuron-specific deletion strategy, our study clearly demonstrates that the postdevelopmental loss of CDKL5 in forebrain excitatory neurons is sufficient to recapitulate many of the structural and behavioral deficits observed in constitutive models. This suggests that the function of CDKL5 extends beyond neurodevelopment and remains crucial for synaptic maintenance and the regulation of complex behaviors in adulthood.

As previously reported in a conditional *Cdkl5* KO model in which selective deletion in excitatory neurons is induced during early cortical development (embryonic day 9.5–10.5 [63]), we observed an increased density of immature dendritic spines accompanied by a marked reduction in mature spines. These findings further support the critical role of CDKL5 in regulating synaptic maturation and stability in the adult brain. Additionally, in *Cdkl5^flox/Y^*(Cre^+^) mice, the overall spine density on CA1 pyramidal neurons was increased compared to that in the controls. However, a higher proportion of these spines exhibited a filopodia-like morphology, characteristic of immature spines. This shift in the balance between immature and mature spines, favoring the former, is also consistent with observations from constitutive *Cdkl5* KO models [69,75], further supporting the idea that CDKL5 function in excitatory neurons is essential to maintain synaptic integrity, and confirming that synaptic abnormalities are not exclusively the result of disrupted neurodevelopment but can also arise following the postnatal loss of CDKL5 function.

The presence of mature spines is essential for the establishment of excitatory synaptic contacts [76]. Accordingly, we observed a reduction in the number of excitatory terminals in *Cdkl5^flox/Y^*(Cre^+^) mice that is consistent with the decreased number of mature spines. In particular, our data show that the postnatal loss of *Cdkl5* in excitatory neurons leads to a reduction in glutamatergic synapse density in both the cortex and hippocampus, without significant changes in the inhibitory synaptic component. The presence of mature spines is essential for the establishment of excitatory synaptic contacts. Therefore, the reduction in the number of excitatory terminals observed in *Cdkl5^flox/Y^*(Cre^+^) mice is consistent with the decreased number of mature spines. Notably, Ricciardi et al. [75], using in the utero electroporation of shRNA targeting *Cdkl5*, similarly reported a decrease in VGLUT1 terminal density in cortical neurons, supporting our findings. However, other studies in the visual cortex [55], and our own recent observations in the somatosensory cortex [57], have reported an increase in VGLUT1 puncta, which appears contradictory and warrants further discussion. This discrepancy could reflect differences in developmental stages, or the timing and method of *Cdkl5* deletion, suggesting a complex and possibly region-specific role of CDKL5 in synapse regulation. Importantly, our model specifically targets *Cdkl5* deletion in excitatory neurons postnatally, whereas many of the contrasting studies use constitutive *Cdkl5* KO mice that lack Cdkl5 in all cell types throughout development. This difference may explain some of the divergent results, as the absence of Cdkl5 in non-excitatory cells (e.g., inhibitory neurons) in constitutive KOs could influence synaptic connectivity in ways that are not captured by excitatory-neuron-specific deletion.

Unlike the reduction observed in excitatory synapses, inhibitory synaptic components remained unchanged in *Cdkl5^flox/Y^*(Cre^+^) mice. Under physiological conditions, axodendritic glutamatergic synapses provide excitatory signals, while axosomatic GABAergic synapses mediate inhibition [77]. Maintaining a proper ratio between these synapse types is essential for normal cortical and hippocampal function. Since inhibitory terminals were unaffected in our model, the ratio of glutamatergic to GABAergic terminals was significantly reduced in both regions, primarily due to the loss of excitatory synapses. This selective vulnerability suggests that CDKL5 plays a critical role in maintaining the delicate balance between excitatory and inhibitory signaling in the brain. Such an imbalance, favoring inhibition, may impair neuronal excitability and disrupt synaptic plasticity. Similar findings have been reported in constitutive *Cdkl5* KO mice, where enhanced inhibitory input was associated with defective long-term potentiation (LTP) [78,79]. Interestingly, a previous study in *Cdkl5* KO models reported alterations in the structure and composition of perineuronal nets [55], extracellular matrix structures that predominantly surround inhibitory interneurons. Since perineuronal nets regulate synaptic stability and limit plasticity, their disruption, particularly involving components such as keratan sulfate [80], could contribute to the altered synaptic balance observed in our model.

Importantly, these findings also suggest that a shift toward increased inhibitory tone in the cortex and hippocampus may contribute to the absence of seizures in this model. Despite previous reports describing spontaneous seizures and increased mortality in this model [61,64], we did not observe overt epileptic activity or premature death in our cohort. Since the genetic background of the mice and the timing of *Cdkl5* deletion closely match previously published conditions, we cannot exclude that environmental and husbandry factors, such as animal management, diet, and environmental stressors, may influence the onset of epileptic seizures and overall survival. This lack of reproducibility has also been reported in other murine models of epilepsy [81], and it nonetheless underscores the limitations of this phenotype in mouse models of CDD. Nevertheless, while dendritic spine loss and synaptic remodeling typically predispose mice to network dysfunction, the preservation of inhibitory input observed here may act as a buffer against hyperexcitability and suppress seizure generation. This protective mechanism resembles what is observed in certain human neuropathologies. For instance, postnatal infants with Down syndrome exhibit a reduced incidence of epilepsy, which has been linked to postnatal dendritic spine loss and an overall enhancement in inhibitory signaling [82].

The presence of microglial activation in the brains of *Cdkl5^flox/Y^*(Cre^+^) mice suggests a potential neuroinflammatory response that is secondary to neuronal dysfunction, a phenomenon previously reported in constitutive *Cdkl5* KO models [59]. Notably, the increase in microglial cells is restricted to the cortex and absent in the hippocampus, indicating a region-specific neuroinflammatory response that may reflect differential vulnerability or the engagement of compensatory mechanisms across brain regions. Since microglial activation is often associated with epilepsy [83,84,85], and considering that this model has shown epileptic activity in other studies [61,64], the elevated microglial cell number may point to underlying neuronal hyperactivity that is not readily detectable without electrophysiological assessment. However, this microgliosis, observed six weeks after *Cdkl5* deletion, does not appear to worsen over time, suggesting that it represents an early and transient response to functional alterations in neuronal circuits rather than a progressive neuroinflammatory process. This pattern implies that microglial activation in this model may reflect a homeostatic adaptation to altered neuronal activity rather than an ongoing degenerative process. Supporting this, the reduced number of neurons observed in the hippocampal CA1 field of *Cdkl5^flox/Y^*(Cre^+^) mice compared to the controls also shows no deterioration over time.

From a phenotypic perspective, *Cdkl5^flox/Y^*(Cre^+^) adult mice display a phenotype that is strikingly similar to that of germline knockouts, including deficits in motor coordination, repetitive and stereotyped behaviors, reduced sociability, and impaired spatial memory. Despite the absence of spontaneous seizures in this model, in contrast with previous reports [61,64], these mice exhibit clear behavioral abnormalities that align with core symptoms of CDD.

Our findings from the postnatal deletion of *Cdkl5* in forebrain glutamatergic neurons reveal a behavioral phenotype that partially overlaps with previously reported studies in [44]. Importantly, unlike the milder phenotype reported in the Nex-Cre conditional KO model [44], which showed relatively selective impairments in hippocampal-dependent memory and modest behavioral alterations, our inducible deletion leads to a more severe and broader spectrum of behavioral deficits. These include pronounced motor coordination impairments and marked social interaction deficits that exceed those observed in the Nex-Cre model. Differently from GABAergic neuron-specific knockouts [43], all conditional models targeting forebrain excitatory neurons, whether during brain development (*Emx1::Cre* [43]; *Nex-Cre* [44]) or in adulthood (present study, *Camk2a-iCre*), consistently display hind-limb clasping behavior. This indicates that the loss of *Cdkl5* in excitatory neurons is sufficient to induce this motor phenotype regardless of the timing of deletion, and further supports the notion that this abnormality is specifically linked to excitatory, rather than inhibitory, neuron dysfunction. All conditional models maintained normal body weight and viability [43,44], paralleling the general health parameters observed in our *Cdkl5^flox/Y^*(Cre^+^) mice.

In contrast, hyperactivity in the open field test and stereotypic jumping behavior, hallmark phenotypes consistently observed in constitutive *Cdkl5* KO mice [69,86], were absent in our *Cdkl5^flox/Y^*(Cre^+^) animals. Similarly, some sensory functions, such as olfaction and mechanical pain perception, remain preserved, likely because the selective deletion of CDKL5 in central glutamatergic neurons spares peripheral sensory neurons and primary sensory pathways. This suggests that CDKL5’s critical role in the adult brain is focused on cortical and hippocampal circuits involved in cognition, memory, and social behavior, rather than on peripheral or primary sensory processing. Indeed, *Cdkl5* is expressed in nociceptive dorsal root ganglia (DRG) neurons [71], further supporting the idea that peripheral CDKL5 expression contributes to pain processing pathways.

Together, these findings emphasize that the timing and cell-type specificity of *Cdkl5* deletion critically influence the spectrum and severity of behavioral phenotypes. While postnatal deletion in forebrain excitatory neurons is sufficient to reproduce core features of CDD, such as motor deficits, social impairments, and cognitive dysfunction, it does not fully recapitulate the broader phenotype observed in constitutive knockouts. This highlights the pivotal role of excitatory neurons in mediating key aspects of the disorder, while also suggesting that additional features may require *Cdkl5* loss in other neuronal populations and/or during early developmental stages.

## 4. Materials and Methods

### 4.1. Animals

All experiments were conducted on male C57BL/6J mice carrying a floxed allele of the *Cdkl5* gene (*Cdkl5^flox/Y^*) either with or without the Camk2a-CreERT2 transgene. *Cdkl5^flox/Y^*–Camk2a-CreERT2 (referred to as *Cdkl5^flox/Y^*(Cre^+^)) mice were generated by crossing *Cdkl5^flox/flox^* females [42] with Camk2a-CreERT2 males [87]. Male *Cdkl5^flox/Y^* littermates lacking the Camk2a-CreERT2 transgene (*Cdkl5^flox/Y^* (Cre^−^)) were used as controls. Postnatal day zero (P0) was designated as the day of birth, and 24 h later, mice were considered to be one-day-old animals (P1). After weaning (P21–23), the mice were housed three to five per cage under a 12 h light/dark cycle in a temperature- and humidity-controlled environment, with food and water provided ad libitum. Animal health and well-being were monitored by the veterinary service. All efforts were made to minimize animal suffering and to keep the number of animals used to a minimum.

The experiments were carried out on a total of 48 mice (*Cdkl5^flox/Y^*(Cre^−^), n = 18; *Cdkl5^flox/Y^*(Cre^+^), n = 30). All animals underwent behavioral testing. Subsets of mice, randomly selected from the behavioral cohort, were used for Western blot analysis (total of n = 6 mice; *Cdkl5^flox/Y^*(Cre^−^) n = 3, *Cdkl5^flox/Y^*(Cre^+^) n = 3) and histological analysis (total of n = 16 mice; *Cdkl5^flox/Y^*(Cre^−^) n = 8, *Cdkl5^flox/Y^*(Cre^+^) n = 8). The exact number of animals used in each experiment is indicated in the corresponding figure legends.

### 4.2. Tamoxifen Treatment

Starting at three months of age, all mice were treated with tamoxifen (Sigma-Aldrich, Saint Louis, MO, USA) dissolved in corn oil (Sigma-Aldrich, Saint Louis, MO, USA) at a concentration of 20 mg/mL. Tamoxifen was administered via intraperitoneal injections at a dose of 100 mg/kg once daily for five consecutive days.

### 4.3. Behavioral Assays

Mice were subjected to a battery of behavioral tests, arranged to minimize the effect of one test on the evaluation of the next. To further reduce inter-test interference, a recovery period of at least 24 h was allowed between each test. All behavioral experiments and subsequent analyses were conducted blind to genotype. Prior to each session, mice were habituated to the testing room for at least 1 h, and all tests were performed at the same time of day.

#### 4.3.1. Marble Burying

The marble burying test [88] was performed by placing mice individually in transparent polycarbonate cages (40 × 25 × 15 cm) filled with 6 cm of clean wood chip bedding. Each mouse was first habituated to the testing cage (without marbles) for 5 min. After the habituation period, the mouse was gently removed, and twenty glass marbles (14.3 mm in diameter) were arranged in a 4 × 5 grid on the surface of the bedding. The mouse was then reintroduced into the same cage and left undisturbed for 30 min. The number of marbles that were at least two-thirds buried at the end of the trial was counted.

#### 4.3.2. Hind-Limb Clasping

Mice were suspended by their tails for 2 min, and their behavior was video-recorded. A clasping event was defined as the retraction of the hind-limbs toward the abdomen and midline. From the video recordings, two parameters were analyzed: (1) the total duration of hind-limb clasping exhibited by each mouse during the 2 min suspension and (2) the percentage of mice showing clasping for at least 3 s during the trial. Evaluation from video recordings was performed by an experimenter blinded to genotype.

#### 4.3.3. Accelerating Rotarod Assay

Motor coordination was assessed using accelerating rotarod apparatus (Ugo Basile, Gemonio, Italy) [89]. Prior to testing, mice were briefly trained on the rotarod at a constant speed of 5 rpm for 30 s. Testing was then performed at an accelerating linear speed (5–35 rpm within 270 s + 30 s max speed). Four testing trials with an intertrial interval of 1 h were performed. The latency to fall from the rotating rod and the number of passive rotations (rotations in which the mouse does not perform any coordinated movement but is passively transported by the rotating apparatus) were recorded for each trial.

#### 4.3.4. Open Field

Mice were individually placed in the center of a square open field arena (50 × 50 cm), and their behavior was recorded for 20 min using a video camera positioned above the center of the arena. Distinct features of locomotor activity, including the total distance traveled, the average locomotion velocity, and the time spent in the central and peripheral zones, were scored using EthoVision XT version 15 (Noldus Information Technology, Wageningen, The Netherlands) [90]. The open field arena was cleaned with 70% ethanol between test subjects.

#### 4.3.5. Three-Chamber Social Interaction Test

Social interaction [91] was assessed using a piece of open-topped plastic apparatus divided into three chambers (20 × 40 × 22 cm) by two transparent walls, each equipped with a retractable doorway to allow access between compartments. The experimental mouse was placed in the center of the middle chamber and allowed to explore the whole apparatus for two trials of 5 min each. In the first trial (habituation phase) the stimulus cages were empty, and the experimental mouse was left undisturbed to explore the apparatus and habituate to the testing environment. At the end of this trial, the experimental mouse was confined to the central chamber using two transparent Plexiglas doors. In the second trial (sociability phase), a stimulus mouse (an age-matched unfamiliar C57BL/6 J male mouse) was introduced into one of the stimulus cages, while a novel object (a plastic cylinder filled with clean wood chip bedding) was introduced in the other one. The position of the social stimulus and of the object was counterbalanced between genotypes. The apparatus and the stimulus cages were cleaned with 70% ethanol at the end of each second trial. The total time spent in each chamber was automatically scored using EthoVision XT version 15 (Noldus Information Technology, Wageningen, The Netherlands).

#### 4.3.6. Barnes Maze

Spatial memory acquisition and retention were evaluated using the Barnes maze test [92]. The apparatus consisted of a circular platform (1 m in diameter) elevated 60 cm above the floor, with twenty evenly spaced holes (each 5 cm in diameter) along the perimeter (Ugo Basile, Gemonio, Italy). An escape box was placed under one of the holes. Mice were trained to locate the escape box over three consecutive days (three trials per day, maximum duration of 3 min per trial), with a 30 min intertrial interval. At the beginning of each trial, mice were placed in the center of the platform under a dark cylindrical chamber for 10 s; the chamber was then lifted to start the trial. Mice that initially failed to locate the escape box within 3 min were gently guided to the target by the experimenter. To assess spatial memory retention, a 90 s probe trial was performed 24 h after the last trial, during which the escape box was removed. Latency to enter the escape box during training sessions, latency to locate the former location of the escape box (i.e., the target zone) during the probe trial, and the total time spent in the target zone were automatically scored using EthoVision XT version 15 (Noldus Information Technology, Wageningen, The Netherlands).

#### 4.3.7. Passive Avoidance

For the passive avoidance test [93], a memory task involving contributions from both the hippocampus and amygdala, the apparatus consisted of a tilting-floor box (47 × 18 × 26 cm) divided into two compartments (illuminated and dark) by a sliding door, and a control unit incorporating a shock generator (Ugo Basile, Gemonio, Italy). On day 1, mice were placed in the illuminated compartment, and upon entering the dark compartment, they received a brief mild foot shock (0.4 mA for 3 s). Mice were removed from the apparatus 15 s after the shock was delivered. After a 24 h retention period, mice were returned to the illuminated compartment, and the latency to re-enter the dark compartment was scored, up to a maximum of 360 s. The two compartments were cleaned with 70% ethanol between the testing of each subject.

#### 4.3.8. Buried-Food Test

The buried-food test assesses olfactory function based on the animal’s ability to detect volatile odors and its natural tendency to use olfactory cues for foraging [94]. The testing protocol consisted of three days and included an odor familiarization phase on day 1, food deprivation on day 2, and testing on day 3. On day 1, mice were placed in a clean cage with a piece of raisin (identical to the one used during the testing day) and left overnight. On day 2, the cages were inspected to confirm raisin consumption, ensuring that the bait was sufficiently palatable. The mice were then food-deprived for 24 h. On day 3, the mice were individually placed in a clean cage containing 4 cm of bedding and allowed to acclimate for 5 min to reduce the interference of novel environment exploration during the test. A piece of raisin was then buried beneath 1 cm of bedding in a randomly selected corner of the cage. Mice were reintroduced into the cage, and the latency to retrieve the raisin using their front paws was measured, up to a maximum of 900 s.

#### 4.3.9. Von Frey Filament Test

Mechanical allodynia was quantified by measuring the hind-paw withdrawal response to von Frey filament stimulation (Ugo Basile, Gemonio, Italy) [95]. Mice were acclimated for 1 h in acrylic cages (12 × 20 × 17 cm) with a wire-grid floor (5 mm^2^). A single, unbending von Frey filament was applied perpendicularly to the plantar surface of each hind-paw with gradually increasing force, and stimulation was stopped upon observation of a withdrawal response. The latency to paw withdrawal and the force (in grams) required to elicit the response were calculated as the average of three measurements taken from each hind-paw.

### 4.4. Histological and Immunohistochemistry Procedures

Animals were anesthetized with isoflurane (2% in pure oxygen) and sacrificed through cervical dislocation. Their brains were quickly removed and cut along the midline. Left hemispheres were Golgi-impregnated or quickly frozen and used for Western blot analyses. Right hemispheres were fixed by immersion in 4% paraformaldehyde in 0.1 M phosphate-buffered saline (PBS) for 48 h, kept in 15–20% sucrose for an additional 24 h, frozen with dry ice, and stored at −80 °C.

#### 4.4.1. Immunofluorescence Staining

Right hemispheres were cut using a freezing microtome (Microm GmbH, Walldorf, Germany) into 30 μm thick coronal sections, which were serially collected in 96-well plates containing a solution composed of 30% glycerol, 30% ethylene glycol, and 0.02% sodium azide in 0.1 M PBS. One out of every eight free-floating sections from the hippocampal formation was incubated overnight at 4 °C with one of the following primary antibodies: rabbit polyclonal anti-AIF-1 antibody (1:300; Thermo Fisher Scientific, Waltham, MA, USA), rabbit polyclonal anti-VGAT antibody (1:300; Synaptic Systems, Göttingen, Germany), or goat polyclonal anti- VGLUT1 antibody (1:300; Synaptic Systems, Göttingen, Germany). The following day, the sections were incubated for 2 h at room temperature with one of the following secondary antibodies: Alexa Fluor 555-conjugated anti-rabbit IgG antibody (1:200; Thermo Fisher Scientific, Waltham, MA, USA), or Alexa Fluor 555-conjugated anti-goat IgG antibody (1:200; Thermo Fisher Scientific, Waltham, MA, USA). Sections were then mounted with DAPI (40,6-diamidino-2-phenylindole)-Fluoromount-G^®^ (SouthernBiotech, Birmingham, AL, USA).

#### 4.4.2. Golgi Impregnation Method

Left hemispheres were Golgi-impregnated using the FD Rapid Golgi StainTM Kit (FD Neuro Technologies, Columbia, MD, USA), as previously described [50]. The hemispheres were cut using a cryostat (Histo-Line Laboratories, Pantigliate, Italy) into 100 µm thick coronal sections, which were directly mounted onto Superfrost^®^ Plus microscope slides (Thermo Fisher Scientific, Waltham, MA, USA) and air-dried at room temperature for 1 day. After drying, the sections were rinsed with distilled water, stained in the developing solution of the FD Rapid Golgi StainTM Kit (FD NeuroTechnologies, Columbia, MD, USA), and coverslipped with DPX mounting medium (Sigma-Aldrich, Saint Louis, MO, USA).

#### 4.4.3. In Situ Hybridization (ISH)

Right hemispheres were cut using a cryostat into 15 μm thick sagittal sections, which were serially collected onto Superfrost^®^ Plus microscope slides (Thermo Fisher Scientific, Waltham, MA, USA). The in situ hybridization (ISH) for *Cdkl5* mRNA was performed using the BaseScope™ technology (Bio-Techne, Minneapolis, MN, USA), following the manufacturer’s protocol. A 1ZZ probe targeting exon 4 of the *CDKL5* transcript was used, as previously described [96].

### 4.5. Image Acquisition and Measurements

Fluorescence images of brain sections processed for AIF-1 immunofluorescence or for *Cdkl5* mRNA detection via BaseScope™ were acquired using a Nikon Eclipse TE 2000-S fluorescence microscope equipped with a DS-Qi2 digital SLR camera (Nikon Instruments Inc., Tokyo, Japan). Fluorescence images of sections immunostained for VGAT or VGLUT1 were acquired using a Nikon Ti-E fluorescence microscope connected to an A1R confocal system (Nikon Instruments Inc., Tokyo, Japan). Bright-field images of Golgi-impregnated brain sections were acquired using a light microscope (Leica Microsystems, Shinjuku City, Tokyo, Japan) equipped with a motorized stage and focus control system, and a color digital camera (Coolsnap-Pro, Media Cybernetics, Rockville, MD, USA).

#### 4.5.1. Quantification of VGAT and VGLUT1 Immunoreactive Puncta

Three to four sections per animal were analyzed. For each section, three images were acquired from layers II–III of the somatosensory cortex and from the stratum oriens of the hippocampus using a 60× oil immersion objective lens (1.32 NA; zoom factor = 8). The density of individual puncta exhibiting VGAT or VGLUT1 immunoreactivity was manually quantified using Image-Pro Plus version 4.5 (Media Cybernetics, Rockville, MD, USA), as previously described [57]. The number of immunoreactive puncta was expressed per µm^2^.

#### 4.5.2. Dendritic Spine Number and Morphology

In Golgi-impregnated sections, dendritic spines located in the basal dendrites of hippocampal pyramidal neurons were captured with a 100× oil immersion objective lens (1.4 NA) and manually counted using Image-Pro Plus version 4.5 (Media Cybernetics, Rockville, MD, USA). For each mouse, 10–12 dendritic segments of 10 μm each were analyzed, and spine density was expressed as the number of spines per 10 μm of dendrite. Based on their morphology, dendritic spines can be classified into two categories reflecting their maturation state: immature spines (filopodium-like, thin, and stubby-shaped) and mature spines (mushroom- and cup-shaped). The number of spines in each category was quantified and expressed as a percentage.

#### 4.5.3. Cell Density

Starting from 20×-magnification images, the number of AIF-1-positive cells in the hippocampus and somatosensory cortex was manually counted using the point tool in Image-Pro Plus version 4.5 (Media Cybernetics, Rockville, MD, USA), and cell density was expressed as AIF-1-positive cells/mm^2^. Similarly, starting from 40×-magnification images, the density of DAPI-positive nuclei in the CA1 field of the hippocampus was manually counted and expressed as cells/mm^2^.

### 4.6. Western Blotting

Tissue samples were homogenized in RIPA buffer (50 mM Tris-HCl, pH 7.4, 150 mM NaCl, 1% Triton-X100, 0.5% sodium deoxycholate, 0.1% SDS) supplemented with 1 mM PMSF and 1% protease and phosphatase inhibitor cocktail (Sigma-Aldrich, Saint Louis, MO, USA). Protein concentration was determined using the Bradford method [97]. Equal amounts of protein (50 μg) were separated by electrophoresis on a 4–12% Mini-PROTEAN^®^ TGX™ Gel (Bio-Rad, Hercules, CA, USA) and transferred to a Hybond ECL nitrocellulose membrane (GE Healthcare, Chicago, IL, USA). The following primary antibodies were used: sheep polyclonal anti-CDKL5 (1:1000; University of Dundee, Dundee, UK); rabbit polyclonal anti-P-EB2 (S222; 1:1000; Covalab, Bron, France); rabbit polyclonal anti-EB2 (1:1000; Abcam, Cambridge, UK); rabbit polyclonal anti-GAPDH (1:5000; Sigma-Aldrich, Saint Louis, MO, USA). The following secondary antibodies were used: HRP-conjugated anti-sheep IgG antibody (1:5000; Jackson ImmunoResearch Laboratories, West Grove, PA, USA); HRP-conjugated anti-rabbit IgG antibody (1:5000; Jackson ImmunoResearch Laboratories, West Grove, PA, USA). A densitometric analysis of digitized Western blot images was performed using the ChemiDocTM MP Imaging System equipped with Image Lab Touch Software (version 3.0.1, Bio-Rad, Hercules, CA, USA), which automatically highlights saturated pixels in red. Images acquired with exposure times that generated protein signals outside of a linear range were not considered for quantification.

### 4.7. Statistical Analysis

Statistical analysis was performed using GraphPad Prism version 8.0.1 (GraphPad Software, Boston, MA, USA). The data are presented as the means ± standard error of the mean (SEM). Normality was assessed using the Shapiro–Wilk test, and the homogeneity of variances using Levene’s test. Depending on assumption testing, Student’s *t*-test or Welch’s *t*-test was used for two-group comparisons. The non-parametric Mann–Whitney U test was used when normality was not met. For multi-factorial analyses, two-way analysis of variance (ANOVA) followed by Fisher’s LSD post hoc test was applied. Outlier data points were excluded using the ROUT method (Q = 1%). The exact sample size (n) for each group is reported in the figure legends. A *p*-value < 0.05 was considered statistically significant.

## 5. Conclusions

Taken together, these results reinforce the idea that CDKL5 remains essential beyond early development for the maintenance of excitatory synapses and the regulation of complex behaviors. Our findings suggest that the postnatal loss of CDKL5 in forebrain excitatory neurons leads to profound alterations in synaptic structure and function, which could mirror those observed in human neurodevelopmental disorders, particularly CDKL5 deficiency disorder (CDD). Notably, the shift toward excessive inhibition, along with the reduction in excitatory synapses, may contribute to the cognitive and motor deficits characteristic of CDD, providing a clearer mechanistic understanding of the disorder.

Moreover, this study underscores the critical need for experimental models that can differentiate between the effects of early and late CDKL5 loss. Such models are key to more accurately recapitulating the human condition of CDD and will enhance our understanding of the ongoing pathophysiological mechanisms in the adult brain. By focusing on late-stage CDKL5 loss, we were able to demonstrate that synaptic dysfunction and behavioral abnormalities persist and potentially evolve throughout adulthood. This highlights the continued role of CDKL5 in the maintenance of synaptic integrity and regulation of complex behaviors even after the completion of neurodevelopment.

In addition, the findings from this study have direct implications for understanding human brain development and the structural malformations seen in CDD. Disruptions in synaptic regulation and maturation, such as the reduction in excitatory synaptic inputs and the loss of dendritic spines, may underlie the cerebral malformations, including reduced brain volume and cortical thinning, observed in affected individuals [98].

This study’s translational relevance extends beyond the basic understanding of CDKL5 in synaptic function, offering insights into the molecular and structural mechanisms that may be at play in human patients with CDD. Future therapeutic strategies aimed at correcting excitatory/inhibitory imbalance or enhancing synaptic maturation could be developed based on these findings. Ultimately, the development of experimental models that more closely reflect human disease will pave the way for identifying novel therapeutic targets and refining potential interventions for neurodevelopmental disorders like CDD.

## Data Availability

The datasets analyzed during the current study are available from the corresponding author upon reasonable request.

## Supplementary Materials

The supporting information can be downloaded at: https://www.mdpi.com/article/10.3390/ijms26146626/s1.
