# Supplementary material for: Adult-Onset Deletion of CDKL5 in Forebrain Glutamatergic Neurons Impairs Synaptic Integrity and Behavior in Mice
Source: Int J Mol Sci. 2025 Jul 10;26(14):6626. doi: 10.3390/ijms26146626 (PMC12294185; doi:10.3390/ijms26146626)
Supplement: Supplementary file 1 [file ijms-26-06626-s001.zip › ijms-3733101-supplementary.pdf]

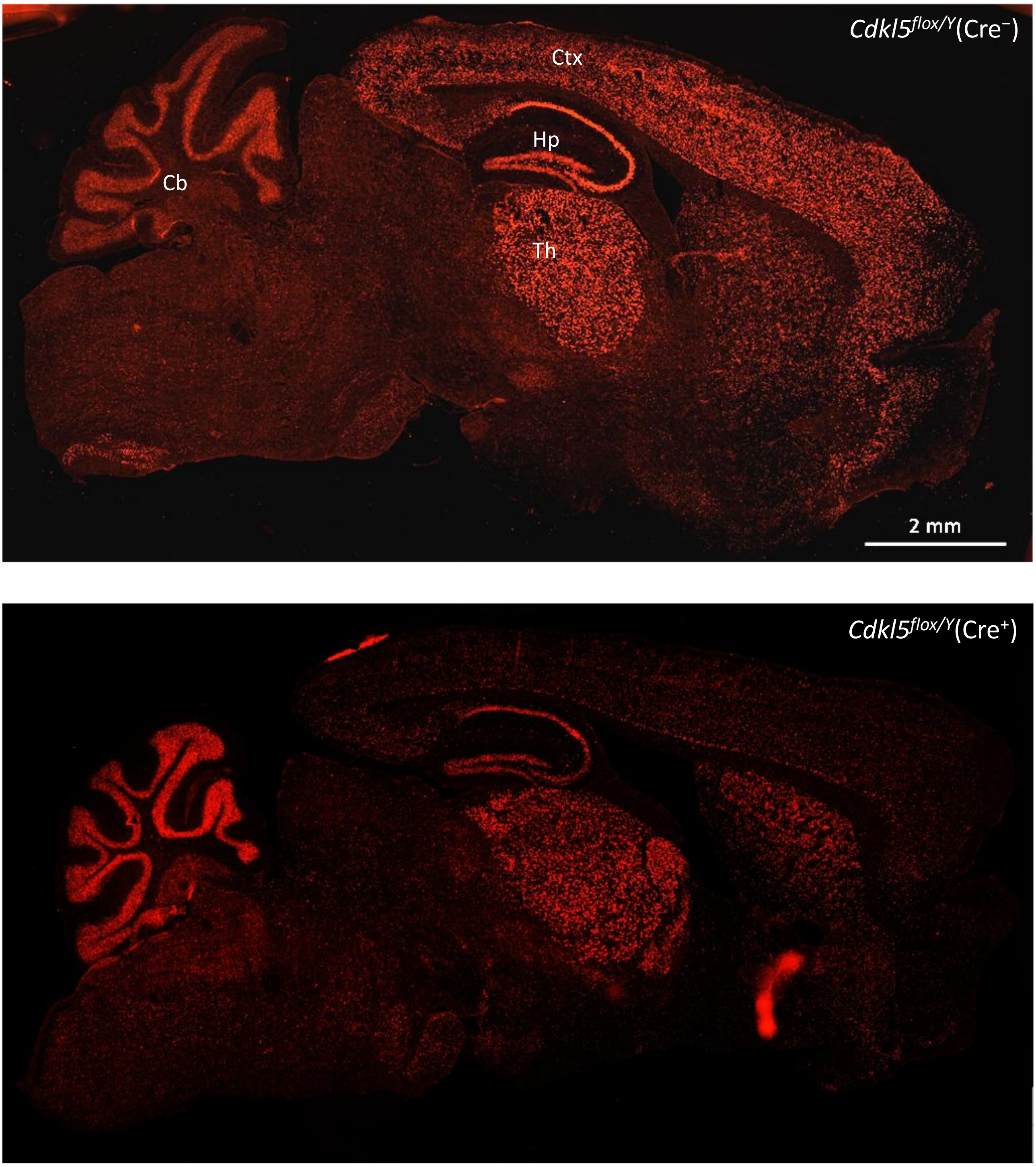


**Figure S1.** *Cdkl5* mRNA expression in the brain of *Cdkl5*$^{flox/Y}$(Cre$^{+}$) mice. Representative sagittal brain sections showing fluorescence in situ hybridization (ISH) for *Cdkl5* mRNA (red) in a *Cdkl5*$^{flox/Y}$(Cre$^{-}$) and *Cdkl5*$^{flox/Y}$(Cre$^{+}$) mouse, six weeks after tamoxifen treatment. Scale bar = 2 mm. Abbreviations: Ctx = somatosensory cortex; Hp = hippocampus; Th = thalamus; Cb = cerebellum.
